# Interfractional change of high-risk CTV D90 during image-guided brachytherapy for uterine cervical cancer

**DOI:** 10.1093/jrr/rrt073

**Published:** 2013-06-03

**Authors:** Yu Ohkubo, Tatsuya Ohno, Shin-ei Noda, Nobuteru Kubo, Akiko Nakagawa, Masahiro Kawahara, Takanori Abe, Hiroki Kiyohara, Masaru Wakatsuki, Takashi Nakano

**Affiliations:** 1 Department of Radiation Oncology, Gunma University Graduate School of Medicine, 3-39-22 Showa-machi, Maebashi, Gunma, 371-8511, Japan; 2 Gunma University Heavy Ion Medical Center, 3-39-22 Showa-machi, Maebashi, Gunma, 371-8511, Japan; 3 Research Center for Charged Particle Therapy, National Institute of Radiological Sciences (NIRS), 4-9-1 Anagawa, Inage-ku, Chiba-shi, 263-8555, Japan

**Keywords:** uterine cervical cancer, radiotherapy, high-dose-rate brachytherapy, 3D image-based planning, dose-volume histogram analysis

## Abstract

The purpose of this study was to evaluate interfractional changes of the minimum dose delivered to 90% of the high-risk clinical target volume (HR-CTV D90) and D2cc of the bladder and rectum during brachytherapy for uterine cervical cancer patients. A total of 52 patients received external beam radiotherapy and high-dose-rate intracavitary brachytherapy (ICBT). For each of four ICBT applications, a pelvic CT scan was performed and the HR-CTV was delineated. Retrospectively, these patients were divided into two groups: (i) the standard dose group with 6 Gy to point A in each ICBT, and (ii) the adaptive dose group with a modified dose to point A to cover the HR-CTV with the 6-Gy isodose line as much as possible. The HR-CTV D90 was assessed in every session, and analyzed as interfractional changes. In the standard dose group, the interfractional changes of the HR-CTV D90 showed a linear increase from the first to the third of the four ICBT (average 6.1, 6.6, 7.0 and 7.1 Gy, respectively). In contrast, those of the adaptive dose group remained almost constant (average 7.2, 7.2, 7.3 and 7.4 Gy, respectively). Especially, in the case of a large HR-CTV volume (≥35 cm^3^) at first ICBT, the total HR-CTV D90 of the adaptive dose group with brachytherapy was significantly higher than that of the standard dose group. There were no significant differences in total D2cc in bladder and rectum between the two groups. Image-guided adaptive brachytherapy based on interfractional tumor volume change improves the dose to the HR-CTV while keeping rectal and bladder doses within acceptable levels.

## INTRODUCTION

Intracavitary brachytherapy (ICBT) plays an important role in uterine cervical cancer treatment. Traditionally, the prescribed dose to point A has been used on the basis of ICRU Report 38 [1]. However, this prescribed dose point based on two orthogonal X-ray films was not able to visualize the cervical tumor, and there has been uncertainty about whether the whole cervical tumor is covered with the prescribed dose. Recently, the dose to the high-risk clinical target volume (HR-CTV) has also been used as one of the indices presenting the dose to the cervical tumor [2, 3]. The HR-CTV is defined as the whole cervix and the presumed extracervical tumor extension at the time of brachytherapy that is a major risk for local recurrence because of residual macroscopic disease. The minimum dose delivered to 90% of the HR-CTV (HR-CTV D90) is considered a good parameter with indications of strong correlation with the regional tumor control rate. Many recent studies have recommended the HR-CTV D90 for controlling cervical tumors [4–6].

The HR-CTV D90, however, of large cervical tumors is often insufficient when using standardized point A in ICBT planning [7]. To improve the dose to the HR-CTV of large tumors, optimized planning based on precise evaluation of tumor size, shape and location [7–9] is important. On the other hand, cervical tumors rapidly decrease in size during external beam radiotherapy (EBRT) and ICBT. The time for 50% tumor regression has been reported as 21 days, occurring after 30.8 Gy with concurrent chemoradiotherapy [10]. Thus, the HR-CTV and its necessary dose would be different in each ICBT even in the same individual. However, as far as we know, changes to the HR-CTV D90 during the treatment time-course in the same patient have not been reported.

In this study, we analyzed the interfractional changes of the HR-CTV D90 in each cervical cancer patient treated with EBRT and ICBT. Additionally, the changes were also analyzed in subgroups based on pretreatment tumor size and the HR-CTV volume at first ICBT.

## MATERIALS AND METHODS

### Patient characteristics

Between October 2008 and October 2010, 61 patients with previously untreated uterine cervical cancer were treated with radiotherapy with radical intent at our institution. In this study, we analyzed the patients who were treated with high-dose-rate intracavity brachytherapy (HDR-ICBT) using a combination of tandem and ovoid applicators. Nine of the patients were ruled out, as one was treated with a vaginal cylinder and eight were treated with interstitial brachytherapy. Therefore, 52 patients were eligible for this analysis (age range, 33–84 years; median, 63 years), and their performance status was 0–2. Patient characteristics are shown in Table 1. All patients underwent MRI of the pelvis for pretreatment evaluation, and cervical tumor dimensions were measured based on T2-weighted images.

**Table 1.** Patient characteristics

	Total	Standard dose group	Adaptive dose group
Number of patients	52	28	24
Staging (FIGO 1994)			
0	2	0	2
IB1	12	6	6
IB2	2	2	0
IIA	4	2	2
IIB	21	13	8
IIIB	11	5	6
Histology			
Squamous cell carcinoma	41	24	17
Adenocarcinoma	6	3	3
Adenosquamous cell carcinoma	1	1	0
Clear cell carcinoma	1	0	1
Carcinoma *in situ*	1	0	1
Unknown	2	0	2
Tumor size in maximum diameter			
≤4 cm (small size)	22	9	13
4–6 cm (medium size)	19	13	6
≥6 cm (large size)	11	6	5
Concurrent chemotherapy			
Weekly cisplatin	21	13	8
Weekly cisplatin + paclitaxel	2	1	1

### Treatment

All patients were treated with a combination of EBRT and HDR-ICBT. In principle, chemotherapy was concurrently combined in patients with tumors > 4 cm in FIGO Stage I–II, in FIGO Stage III–IVA, or with pelvic lymph-node metastases. The exclusion criteria for chemotherapy were age over 75 years or severe concomitant diseases. Of the 52 patients, 23 (44%) were treated with concurrent chemoradiotherapy: 21 patients with weekly cisplatin at 40 mg/m^2^ (2–5 times), and two patients with weekly cisplatin at 30 mg/m^2^ and paclitaxel at 50 mg/m^2^ (6 times).

External beam radiotherapy to the pelvis was performed with anterior and posterior parallel-opposed portals or the box technique using 10-MV X-rays. The common field borders were at the L4/5 intervertebral space superiorly, at the inferior border of the obturator foramen inferiorly, and 1.5–2.0 cm lateral to the inner bony margins of the true pelvis. A total of 50 Gy was delivered to the pelvic sidewall, at 2.0 Gy a day, 5 days a week. A midline block (3-cm width at the isocenter) was inserted after delivering 20 or 30 Gy to the whole pelvis.

HDR-ICBT was performed using the ^192^Ir remotely-controlled-after-loading system (microSelectron HDR, Nucletron, Veenendaal, The Netherlands). The iridium source strength ranged between 185 and 370 GBq (5–10 Ci). A Foley catheter was inserted and ballooned with 7 ml of contrast medium to localize the bladder neck. The bladder was filled with 100 ml of normal saline to avoid high-dose irradiation to its whole volume. Vaginal packing was done to reduce the rectum-irradiated dose.

Both a C-arm type X-ray unit and a CT scanner were installed in the treatment room. After implantation of the applicators, two orthogonal X-rays were taken, followed by a plain pelvic CT scan performed in the same supine position on the same treatment couch. A series of transverse CT images with 3-mm slice thickness were obtained with the applicators in place. The CT image datasets were imported into the treatment-planning system (Plato Brachytherapy Planning System, Nucletron). Then, delineation of the target volumes and organs at risk (OARs) was performed. Following the GEC-ESTRO recommendations for 3D image-based brachytherapy for cervical cancer [2, 3], the HR-CTV and the external walls of the bladder and rectum were delineated. For precise delineation, reference was always made to the MRI images at diagnosis and just before the first HDR-ICBT. HDR-ICBT was performed once a week, concurrently with central-shielding EBRT.

### CT-based 3D dose-volume evaluation

All contouring (HR-CTV, bladder and rectum) was reviewed by two radiation oncologists (Y.O. and T.O.). Cumulative dose–volume histograms (DVHs) were calculated for the HR-CTV and the OARs. The minimum doses delivered to 2 cm^3^ (D2cc) of the most irradiated volumes of the bladder and rectum were derived from the DVH according to the GEC-ESTRO recommendation [3]. From DVH analysis, all parameters of the HR-CTV volume, HR-CTV D90, D2cc of bladder, and D2cc of rectum were obtained for four fractions of HDR-ICBT for each patient. We analyzed these changes in three subgroups based on tumor size: small tumor, ≤4 cm in maximum diameter in MRI images at pretreatment; medium tumor, 4–6 cm; large tumor, ≥6 cm. Similarly, we analyzed two subgroups based on the HR-CTV volume at first ICBT: small group, <35 cm^3^ in HR-CTV volume; large group, ≥ 35 cm^3^. The reason for setting the cut-off value at 35 cm^3^ was that the HR-CTV volume is estimated to be ∼ 35 cm^3^ in the case of 4-cm tumors of maximum diameter, assuming the cervical tumor to be a spherical object.

The doses to the HR-CTV and OARs from both EBRT and HDR-ICBT were normalized to biologically equivalent doses in 2-Gy fractions (Gy_EQD2_) based on the linear-quadratic model: the α/β value of ‘10’ for the HR-CTV, and the α/β value of ‘3’ for late-responding tissues (OARs). In the standard dose group, the combined prescribed dose of whole-pelvic EBRT and all ICBT to point A was 52–62 Gy_EQD2_ (20–30 Gy/10–15 fractions of EBRT plus 24 Gy/4 fractions of ICBT). In principle, D2cc of the rectum was, as recommended, below 6 Gy in every HDR-ICBT. Further, the dose restrictions of the combined dose of whole-pelvic EBRT and all ICBT for D2cc of the bladder and rectum are as follows: 85 Gy_EQD2_ for the bladder and 75 Gy_EQD2_ for the rectum.

### Standard dose group and adaptive dose group

Retrospectively, these patients were divided into two groups. Of the 52 patients in the study, 28 were prescribed 6 Gy per fraction at point A in each ICBT for four fractions with the standard loading pattern of our practice (‘standard dose group’). In this group, there was no dose modification for OARs, as the intestinal dose achieved our dose constraints based on visual inspection of the isodose lines to the intestine.

**Fig. 1.**
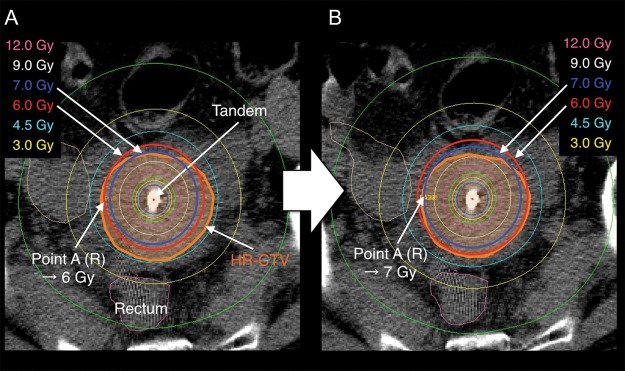
Axial computed tomography image of an example case of ICBT. (**A**) A dose of 6 Gy was administered to point A. The high-risk clinical target volume (HR-CTV) was not covered with the 6-Gy isodose line. (**B**) A dose of 7 Gy was prescribed to point A. The 6-Gy isodose line almost encompasses the HR-CTV.

The other 24 patients were treated with a modified prescribed dose to point A. These patients were classified as the ‘adaptive dose group’. In this group, the prescribed point A dose was increased or decreased so as to fit the 6-Gy isodose line to the HR-CTV as much as possible. In principle, dose adaptation to point A was determined by the attending doctor at ICBT, as quantitative criteria had not yet been established. Figure 1A shows an example case of a large HR-CTV not being encompassed by the 6-Gy isodose line when a dose of 6 Gy is administered to point A. If the point A dose is increased to 7 Gy, the HR-CTV is well covered with the 6-Gy isodose line (Fig. 1B). When the tumor had shrunk through the course of each HDR-ICBT, the prescription dose to point A could be decreased to the extent that the HR-CTV could be covered with the 6-Gy isodose line. The prescribed dose to point A was determined with every effort to minimize the dose to the OARs (the bladder and rectum).

### Statistical analysis

An unpaired Mann–Whitney U test was used to compare the dose between the ‘standard dose’ and ‘adaptive dose’ groups. All *P* values reported were two-sided; *P* < 0.05 was considered statistically significant.

## RESULTS

### Interfractional change in the HR-CTV D90

**Fig. 2.**
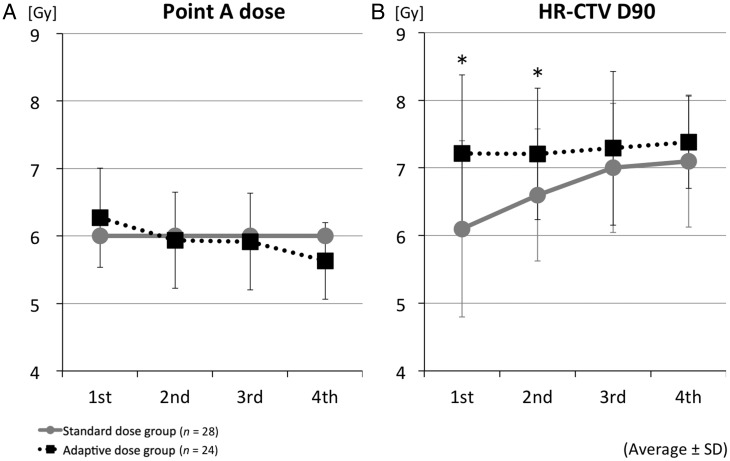
Changes during treatment time-course of prescribed point A dose (**A**), and the HR-CTV D90 (**B**) of first to fourth HDR-ICBT. In the standard dose group, the first and second HR-CTV D90 were significantly lower than those of the adaptive dose group. Circles and solid lines show the averages for the standard dose group; boxes and dotted lines show the averages for the adaptive dose group. Bars indicate standard deviations. Asterisks indicate *P* < 0.05 by Mann–Whitney U test.

Figure 2 shows the changes over time of the point A dose and the HR-CTV D90 from the first to fourth HDR-ICBT. In the standard dose group, the point A dose was always 6 Gy. On the other hand, the HR-CTV D90 increased through all four courses of ICBT, especially from the first to third ICBT: 6.1 Gy on average (SD = 1.3) in the first ICBT, 6.6 Gy (SD = 1.0) in the second, 7.0 Gy (SD = 1.0) in the third, and 7.1 Gy (SD = 1.0) in the fourth. The total HR-CTV D90 of EBRT and ICBT was 65.0 Gy_EQD2_ on average (SD = 7.3).

In the adaptive dose group, the average point A dose decreased with time, as shown in Fig. 2A: 6.3 Gy (SD = 0.7) in the first ICBT, 5.9 Gy (SD = 0.7) in the second, 5.9 Gy (SD = 0.7) in the third, and 5.6 Gy (SD = 0.6) in the fourth. Conversely, the HR-CTV D90 in the adaptive dose group was almost constant throughout ICBT: 7.2 Gy (SD = 1.2) in the first ICBT, 7.2 Gy (SD = 1.0) in the second, 7.3 Gy (SD = 1.1) in the third, and 7.4 Gy (SD = 0.7) in the fourth. The differences in HR-CTV D90 were statistically significant between the standard dose and adaptive dose groups in the first and second ICBT (*P* = 0.003 and 0.029, respectively). The total HR-CTV D90 of EBRT and ICBT was 68.9 Gy_EQD2_ on average (SD = 13.0) and had a higher tendency in the adaptive dose group compared with the standard dose group, although this was not statistically significant (*P* = 0.053).

### Interfractional change by pretreatment tumor size

**Fig. 3.**
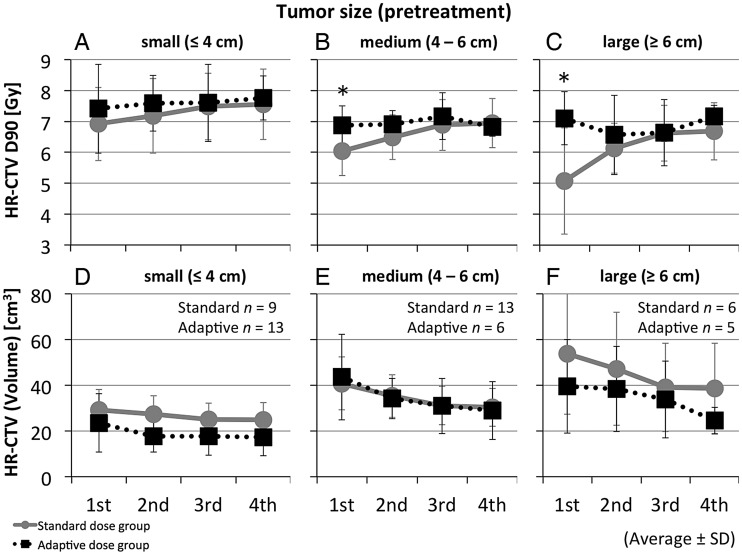
Changes in the HR-CTV D90 and volume during treatment time-course in differentiated pretreatment tumor size groups. A small group (≤4 cm) is shown in (**A**) and (**D**), a medium group (4–6 cm) in (**B**) and (**E**), and a large group (≥6 cm) in (**C**) and (**F**). At first ICBT, the HR-CTV D90 in the standard dose group was significantly lower than in the adaptive dose group for the medium and large tumor groups. Circles and solid lines show averages for the standard dose group; boxes and dotted lines show averages for the adaptive dose group. Bars indicate standard deviations. Asterisks indicate *P* < 0.05 by Mann-Whitney U test.

Figure 3 shows the interfractional changes over time of the HR-CTV D90 and HR-CTV volume, presented in each pretreatment tumor size group (small, medium, and large). In the medium (4–6 cm) and large (≥6 cm) tumor groups, the values of the HR-CTV D90 at first ICBT in the standard dose group were significantly lower than those in the adaptive dose group (*P* = 0.035 and *P* = 0.017, respectively). In each size group the total HR-CTV D90 of EBRT and ICBT tended to be higher in the adaptive dose group than in the standard dose group, although the differences were not statistically significant. Regarding the volume of the HR-CTV, the small and large tumor groups showed relatively lower volumes in the adaptive dose group compared with the standard dose group, although there was no statistical significance indicated.

### Interfractional change by the HR-CTV volume at first ICBT

**Fig. 4.**
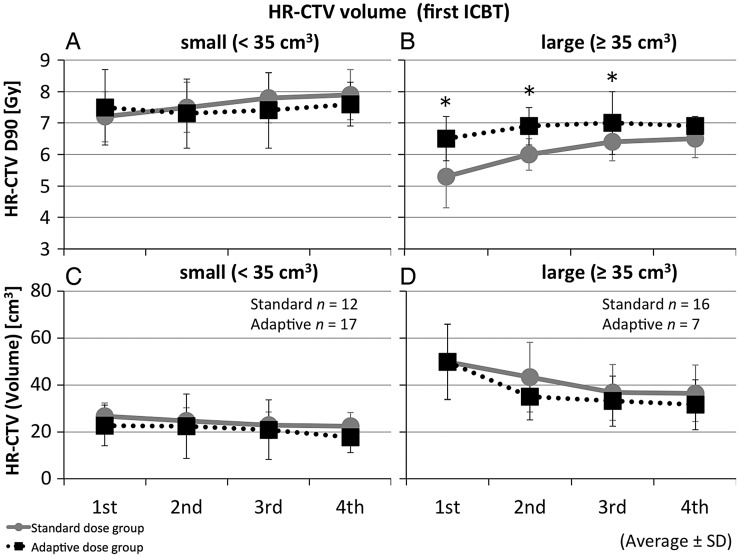
Changes in the HR-CTV D90 and volume during treatment time-course in differentiated HR-CTV volumes at first ICBT. The ‘small’ group (<35 cm^3^) is shown in (**A**) (**C**) and the ‘large’ group (≥35 cm^3^) in (**B**) (**D**). At first, second and third ICBT, the HR-CTV D90 in the standard dose group was significantly lower than in the adaptive dose group in the large tumor groups. In the adaptive dose group, the HR-CTV volume at second ICBT diminished more steeply in the large tumor groups. Circles and solid lines show averages for the standard dose group; boxes and dotted lines show averages for the adaptive dose group. Bars indicate standard deviations. Asterisks indicate *P* < 0.05 by Mann–Whitney U test.

Figure 4 shows the interfractional changes of the HR-CTV D90 and volume, presented in each HR-CTV volume at first ICBT (small and large). In the large tumor group (≥35 cm^3^), the HR-CTV D90 values at first, second and third ICBT in the adaptive dose group were significantly higher than those in the standard dose group (*P* = 0.008, 0.004 and 0.037, respectively). The total HR-CTV D90 of EBRT and ICBT in the large tumor groups was 61.5 Gy_EQD2_ on average (SD = 5.6) in the standard dose group, and 71.8 Gy_EQD2_ (SD = 8.0) in the adaptive dose group. There was a significant difference between the two groups (*P* = 0.005). On the other hand, in the smaller group (<35 cm^3^), the changes with time were minor during the four fractions of ICBT, and there were no significant differences between the adaptive and standard dose groups.

### Interfractional changes in D_2cc_ of rectum and bladder

**Fig. 5.**
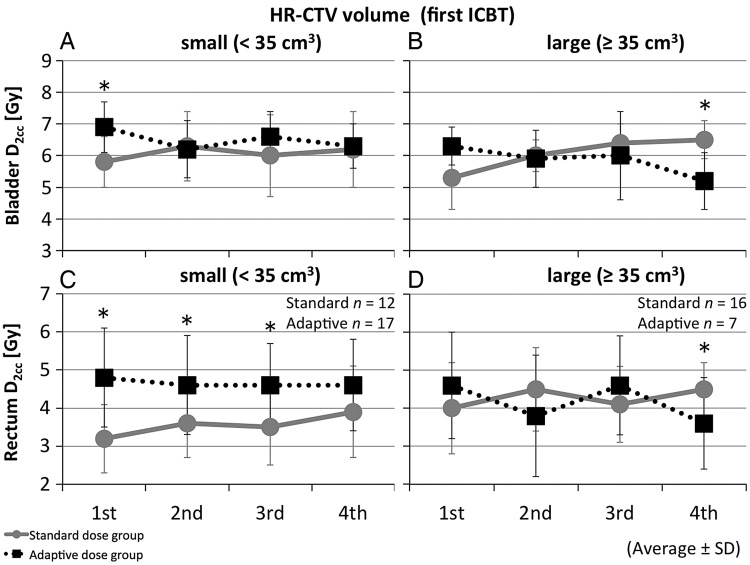
Changes in D2cc of bladder and rectum during treatment time-course in differentiated HR-CTV volumes at first ICBT. A small group (<35 cm^3^) is shown in (**A**) and (**C**) and a large group (≥35 cm^3^) in (**B**) and (**D**). Circles and solid lines show averages for the standard dose group; boxes and dotted lines show averages for the adaptive dose group. Bars indicate standard deviations. Asterisks indicate *P* < 0.05 by Mann–Whitney U test.

Figure 5 shows the interfractional changes of rectal and bladder D2cc by HR-CTV volume at first ICBT. In the small tumor group (< 35 cm^3^), D2cc of bladder at first ICBT was significantly higher in the adaptive dose group (*P* = 0.001). Also, the D2cc values for the rectum from first to third ICBT were significantly higher in the adaptive dose group (*P* = 0.002, 0.023, and 0.009, respectively), although the doses were kept below 6 Gy in most cases. On the other hand, in the large tumor group (≥35 cm^3^), D2cc of bladder and rectum at fourth ICBT in the adaptive group were significantly lower (*P* = 0.041 and *P* = 0.021, respectively).

In the small tumor group (<35 cm^3^), the total D2cc values of bladder and rectum were 70.3 Gy_EQD2_ on average (SD = 14.1) and 44.6 Gy_EQD2_ (SD = 9.1) in the standard dose group, respectively, while the corresponding values in the adaptive dose group were 73.2 Gy_EQD2_ (SD = 16.3) and 52.6 Gy_EQD2_ (SD = 19.3), respectively. In the large tumor group (≥35 cm^3^), the total D2cc values of bladder and rectum in the standard dose group were 72.7 Gy_EQD2_ on average (SD = 9.1) and 54.3 Gy_EQD2_ (SD = 8.1), respectively, while the corresponding values in the adaptive dose group were 75.1 Gy_EQD2_ (SD = 8.4) and 56.6 Gy_EQD2_ (SD = 10.8), respectively. The differences of total D2cc in bladder and rectum were not significant between the standard and adaptive dose groups in either of the two tumor volume groups.

## DISCUSSION

In the current study, we demonstrated the interfractional changes in the HR-CTV D90 and D2cc of rectum and bladder in HDR-ICBT. At first and second HDR-ICBT, the HR-CTV D90 in the adaptive dose group was significantly higher than in the standard dose group (Fig. 2B). In the standard dose group, insufficient doses might have been administered to the HR-CTV at first and second HDR-ICBT, although the HR-CTV D90 had increased linearly with regression of the tumor during first to third ICBT. On the other hand, the treatment strategy in the adaptive dose group was to fit the 6-Gy isodose line to the HR-CTV as much as possible. In this manner, the point A dose at first ICBT was a little higher in the adaptive dose group, and then, the values slowly decreased during the course of ICBT (Fig. 2A). As a result, the total dose of both external irradiation and HDR-ICBT was higher in the adaptive dose group (68.9 Gy_EQD2_ vs 65.0 Gy_EQD2_), although there was no statistical significance.

We also analyzed HR-CTV D90 changes by pretreatment tumor size and HR-CTV volume at first ICBT. When differentiated by pretreatment tumor size, the HR-CTV D90 was nearly comparable between the standard and adaptive dose groups for small tumors (≤4 cm). But for the medium (4–6 cm) and large (≥6 cm) tumor groups, the HR-CTV D90 in the first ICBT of the standard dose group was significantly lower than that of the adaptive dose group (Fig. 3). These results suggested the following: if MRI examination before treatment revealed a tumor size > 4 cm, adaptive therapy would be the better treatment option because of the good coverage to the HR-CTV D90. Otherwise, in the large tumor groups, each volume in the adaptive dose group was lower than the standard dose group over ICBT. This result indicated that the extent of tumor shrinking during EBRT (before ICBT) could vary widely.

When differentiated by HR-CTV volumes at first ICBT, the volume at second ICBT in the adaptive dose group declined more steeply in the large tumor group (≥35 cm^3^) (Fig. 4). Based on this, HR-CTV D90 values in the adaptive dose group were significantly higher during first to third ICBT in the large tumor cases, and the total HR-CTV D90 values were statistically higher than in the standard dose group. Therefore, adaptive ICBT planning seemed to have a major advantage in the patients with large tumors at first ICBT (≥35 cm^3^ in HR-CTV volume).

The rectal D2cc of the adaptive dose group was significantly higher from first to third ICBT than that of the standard dose group for small tumors (<35 cm^3^ at first ICBT). But in total equivalent doses there were no significant differences between the adaptive and standard dose groups in any of the tumor size groups. In addition, the total D2cc of rectum in the adaptive dose group was 52.6 Gy_EQD2_ for small tumors and 56.6 Gy_EQD2_ for large tumors. These doses kept our dose constraints and were considered to be within an acceptable range based on recently reported thresholds [11, 12]. This is mainly because dwell positions and dwell times of the source were fine-tuned using the 3D treatment-planning system: visualization of the HR-CTV and the isodose line. Further, due to higher doses to the HR-CTV in the adaptive dose group in early ICBT sessions [especially in patients with large tumors (≥35 cm^3^)], rapid tumor shrinkage might allow delivery of high doses to the HR-CTV without increasing the doses to OARs in the following sessions of brachytherapy. Sufficient vaginal packing is also important to reduce the rectal wall dose, especially with tumor size decreasing in response to treatment. It is essential to balance the trade-off relationship between OAR dose and HR-CTV D90, increasing HR-CTV D90 as much as possible within tolerable OAR dose limits. But when large tumors lean to one side or the other in the cervix, it is difficult to cover the HR-CTV with enough doses only using tandem and ovoids. In this situation, the rectum and bladder must be irradiated with much higher doses. Several recent studies have reported new techniques of combined intracavitary and interstitial brachytherapy [13–15]. These techniques have the potential to increase the dose to the HR-CTV within acceptable dose limits to OARs, especially in cases of irregularly shaped, huge or eccentrically located tumors.

In this study, we used CT images in each image-guided brachytherapy (IGBT); MRI images at diagnosis and just before first ICBT were used as references for precise delineation. MRI-based treatment planning for each fraction may be an ideal approach, but the lack of MRI availability in many centers has placed limits on its use in Japan. Moreover, its use is also limited because it is at present not covered by the health insurance system. Research by the American Brachytherapy Society has revealed that most centers in America currently use CT images, not MRI images [16]. MRI is a better modality for precise delineation of both the cervix and residual disease, whereas CT scanning approaches have some uncertainty in defining the HR-CTV [17]. Meanwhile, a method similar to ours, feasible for IGBT and using MRI for the first ICBT session, followed by CT-based planning for subsequent fractions, has been reported [18, 19].

## CONCLUSION

In conclusion, this study revealed that dose coverage of the HR-CTV with the fixed-dose prescription of 6 Gy × 4 fractions to point A would become insufficient, especially for cases with medium (4–6 cm) and large tumors (≥6 cm) before treatment, or large HR-CTV volume (≥35 cm^3^) at first ICBT. On the other hand, adaptive dose prescription to the HR-CTV could accomplish better dose coverage of the HR-CTV, with an allowable dose range to the OARs. Further study is needed to clarify whether or not these achievements are associated with improvements in local tumor control rate without increasing toxicities.
